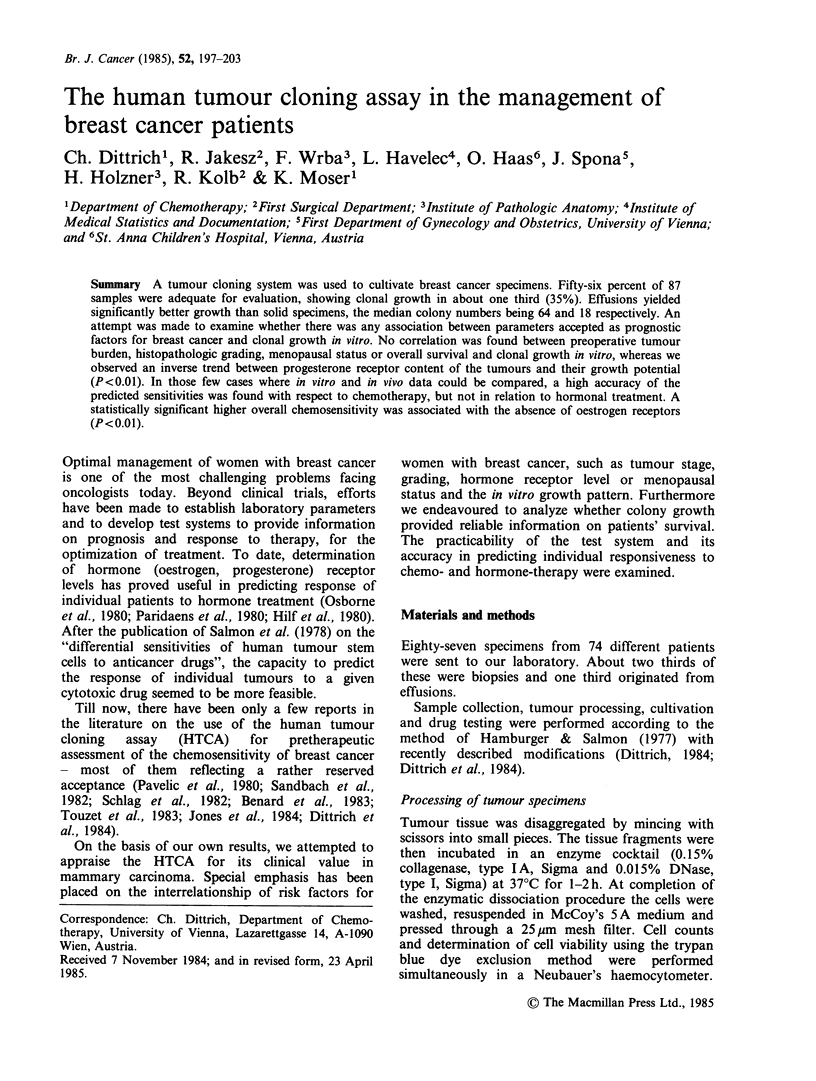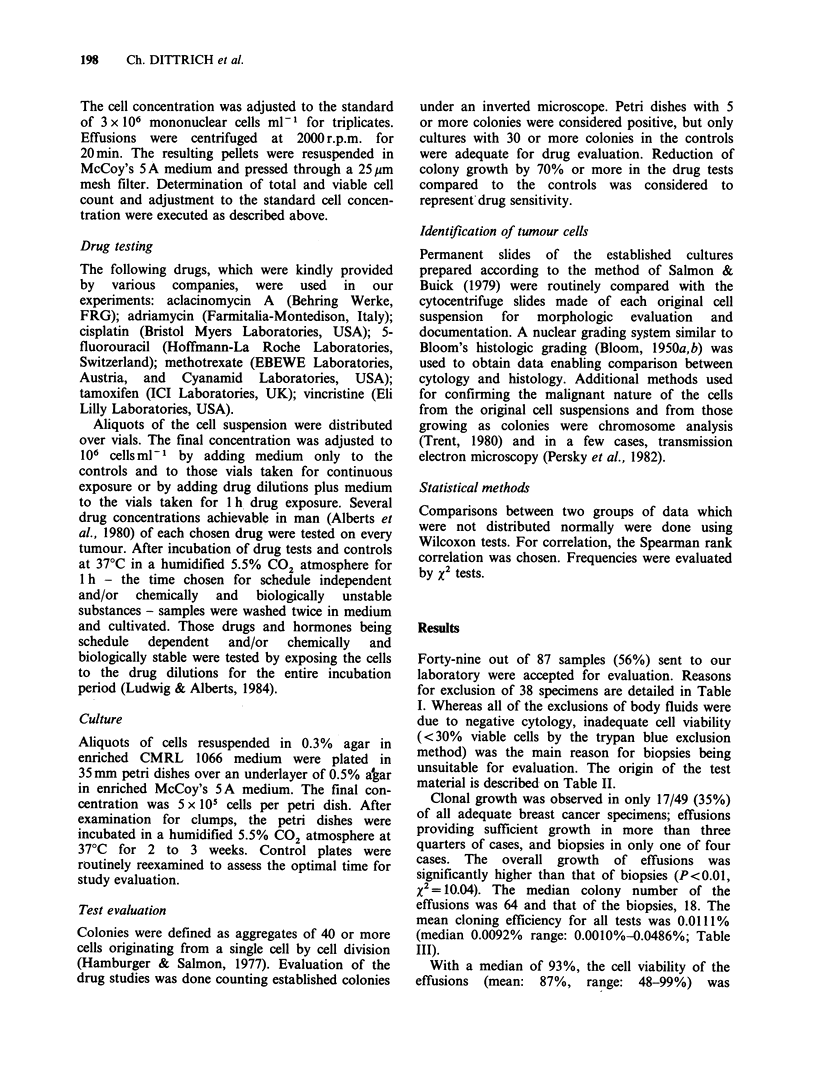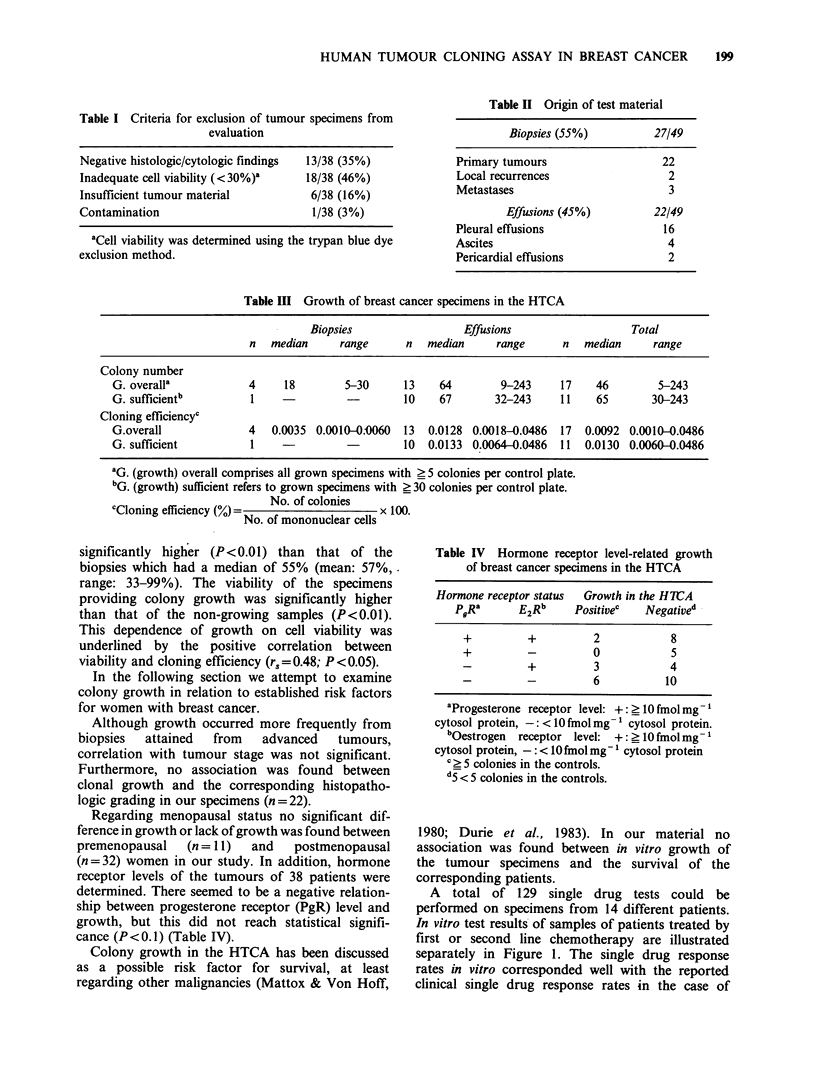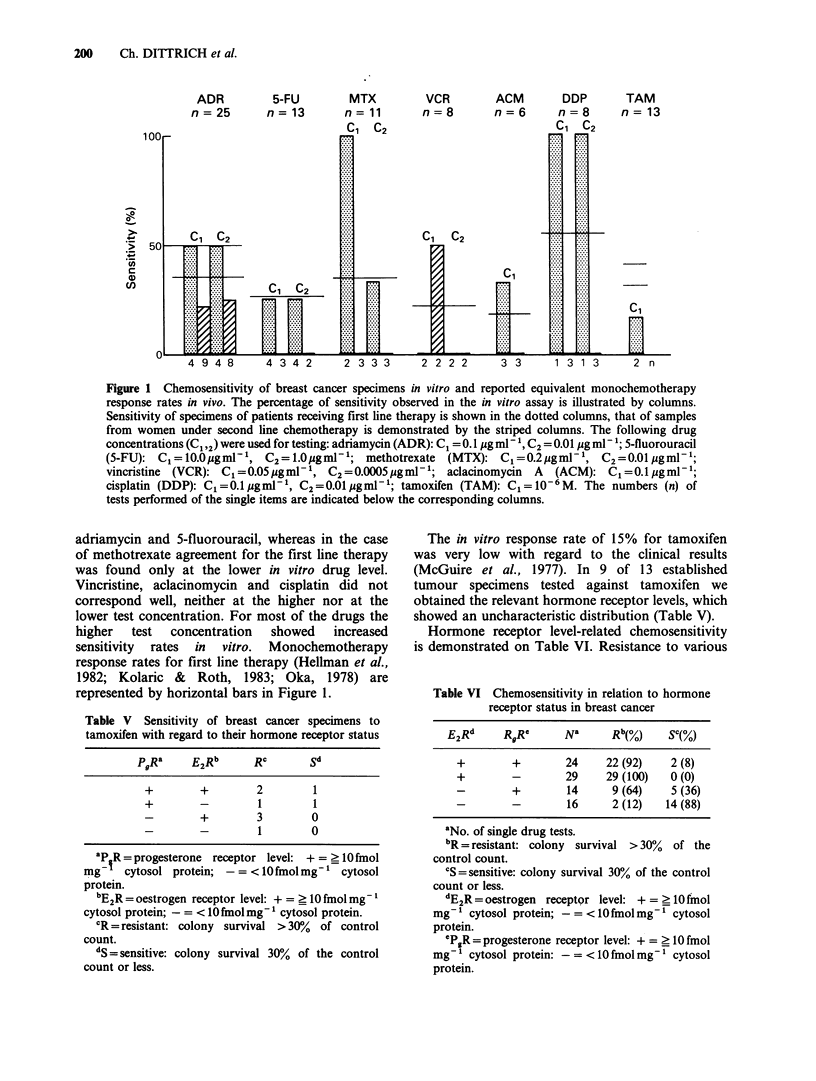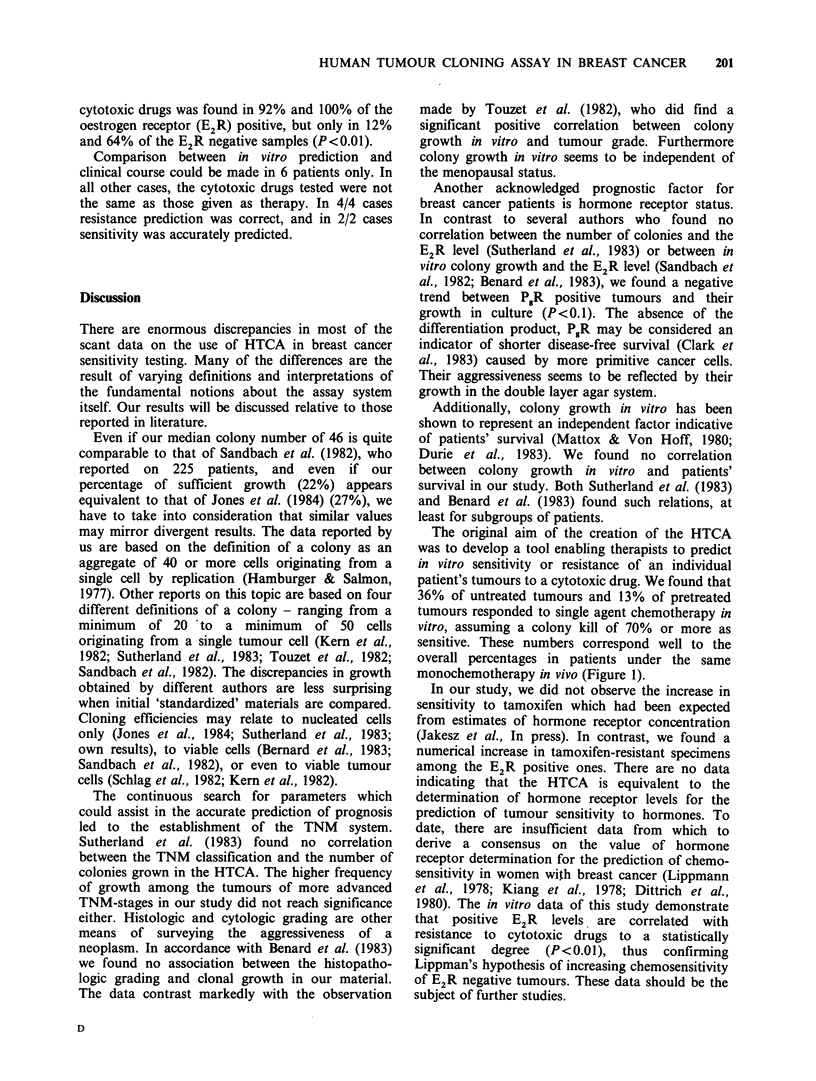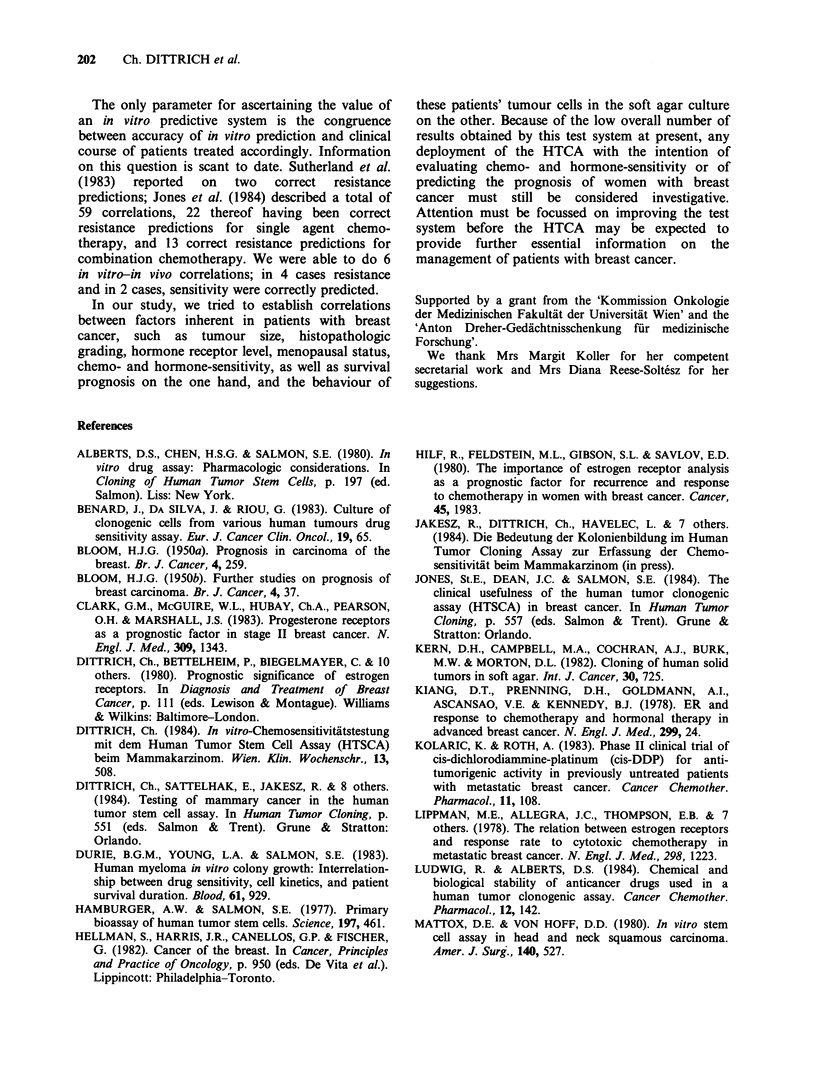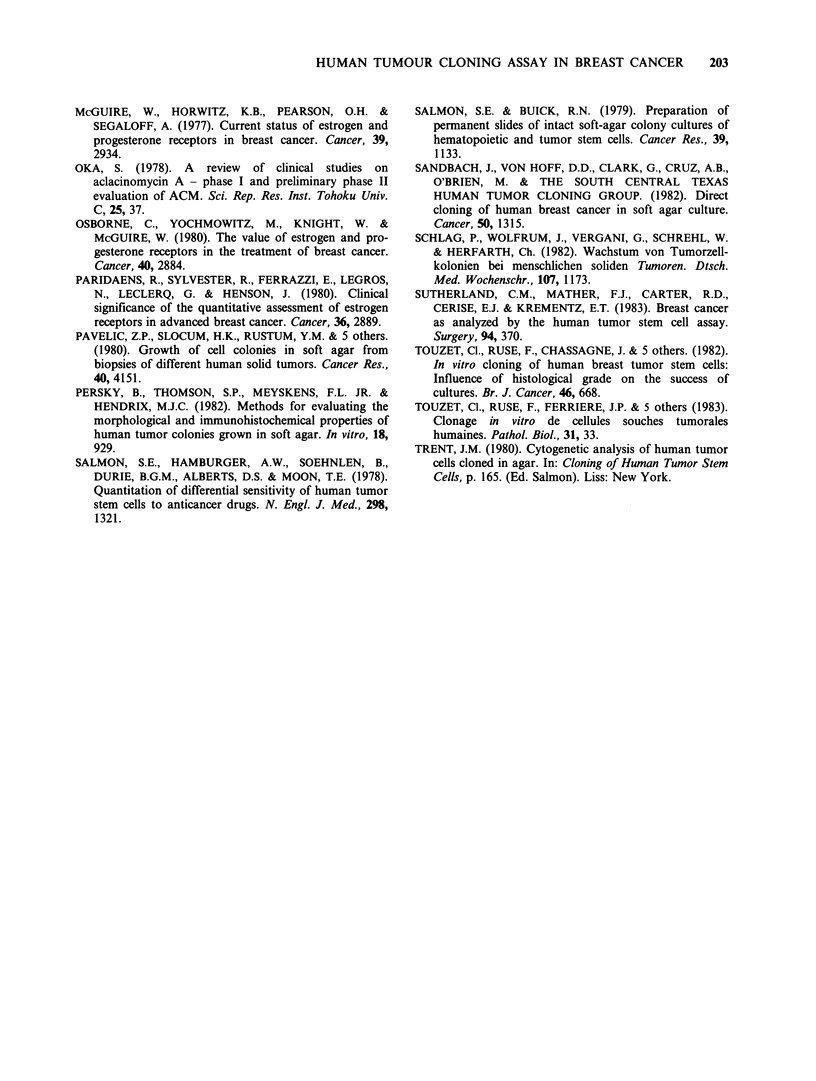# The human tumour cloning assay in the management of breast cancer patients.

**DOI:** 10.1038/bjc.1985.178

**Published:** 1985-08

**Authors:** C. Dittrich, R. Jakesz, F. Wrba, L. Havelec, O. Haas, J. Spona, H. Holzner, R. Kolb, K. Moser

## Abstract

A tumour cloning system was used to cultivate breast cancer specimens. Fifty-six percent of 87 samples were adequate for evaluation, showing clonal growth in about one third (35%). Effusions yielded significantly better growth than solid specimens, the median colony numbers being 64 and 18 respectively. An attempt was made to examine whether there was any association between parameters accepted as prognostic factors for breast cancer and clonal growth in vitro. No correlation was found between preoperative tumour burden, histopathologic grading, menopausal status or overall survival and clonal growth in vitro, whereas we observed an inverse trend between progesterone receptor content of the tumours and their growth potential (P less than 0.01). In those few cases where in vitro and in vivo data could be compared, a high accuracy of the predicted sensitivities was found with respect to chemotherapy, but not in relation to hormonal treatment. A statistically significant higher overall chemosensitivity was associated with the absence of oestrogen receptors (P less than 0.01).


*Br. J. Cancer* (1985), **52**, 197–203

### The human tumour cloning assay in the management of breast cancer patients

Ch. Dittrich[1], R. Jakesz[2], F. Wrba[3], L. Havelec[4], O. Haas[6], J. Spona[5], H. Holzner[3], R. Kolb[2] & K. Moser[1]

[1]*Department of Chemotherapy;* [2]*First Surgical Department;* [3]*Institute of Pathologic Anatomy;* [4]*Institute of Medical Statistics and Documentation;* [5]*First Department of Gynecology and Obstetrics, University of Vienna; and* [6]*St. Anna Children's Hospital, Vienna, Austria*

**Summary** A tumour cloning system was used to cultivate breast cancer specimens. Fifty-six percent of 87 samples were adequate for evaluation, showing clonal growth in about one third (35%). Effusions yielded significantly better growth than solid specimens, the median colony numbers being 64 and 18 respectively. An attempt was made to examine whether there was any association between parameters accepted as prognostic factors for breast cancer and clonal growth *in vitro*. No correlation was found between preoperative tumour burden, histopathologic grading, menopausal status or overall survival and clonal growth *in vitro*, whereas we observed an inverse trend between progesterone receptor content of the tumours and their growth potential ($P<0.01$). In those few cases where *in vitro* and *in vivo* data could be compared, a high accuracy of the predicted sensitivities was found with respect to chemotherapy, but not in relation to hormonal treatment. A statistically significant higher overall chemosensitivity was associated with the absence of oestrogen receptors ($P<0.01$).

Optimal management of women with breast cancer is one of the most challenging problems facing oncologists today. Beyond clinical trials, efforts have been made to establish laboratory parameters and to develop test systems to provide information on prognosis and response to therapy, for the optimization of treatment. To date, determination of hormone (oestrogen, progesterone) receptor levels has proved useful in predicting response of individual patients to hormone treatment (Osborne *et al.*, 1980; Paridaens *et al.*, 1980; Hilf *et al.*, 1980). After the publication of Salmon *et al.* (1978) on the "differential sensitivities of human tumour stem cells to anticancer drugs", the capacity to predict the response of individual tumours to a given cytotoxic drug seemed to be more feasible.

Till now, there have been only a few reports in the literature on the use of the human tumour cloning assay (HTCA) for pretherapeutic assessment of the chemosensitivity of breast cancer – most of them reflecting a rather reserved acceptance (Pavelic *et al.*, 1980; Sandbach *et al.*, 1982; Schlag *et al.*, 1982; Benard *et al.*, 1983; Touzet *et al.*, 1983; Jones *et al.*, 1984; Dittrich *et al.*, 1984).

On the basis of our own results, we attempted to appraise the HTCA for its clinical value in mammary carcinoma. Special emphasis has been placed on the interrelationship of risk factors for women with breast cancer, such as tumour stage, grading, hormone receptor level or menopausal status and the *in vitro* growth pattern. Furthermore we endeavoured to analyze whether colony growth provided reliable information on patients' survival. The practicability of the test system and its accuracy in predicting individual responsiveness to chemo- and hormone-therapy were examined.

Correspondence: Ch. Dittrich, Department of Chemotherapy, University of Vienna, Lazarettgasse 14, A-1090 Wien, Austria.

Received 7 November 1984; and in revised form, 23 April 1985.

### Materials and methods

Eighty-seven specimens from 74 different patients were sent to our laboratory. About two thirds of these were biopsies and one third originated from effusions.

Sample collection, tumour processing, cultivation and drug testing were performed according to the method of Hamburger & Salmon (1977) with recently described modifications (Dittrich, 1984; Dittrich *et al.*, 1984).

### *Processing of tumour specimens*

Tumour tissue was disaggregated by mincing with scissors into small pieces. The tissue fragments were then incubated in an enzyme cocktail (0.15% collagenase, type I A, Sigma and 0.015% DNase, type I, Sigma) at 37°C for 1–2 h. At completion of the enzymatic dissociation procedure the cells were washed, resuspended in McCoy's 5 A medium and pressed through a 25 $\mu$m mesh filter. Cell counts and determination of cell viability using the trypan blue dye exclusion method were performed simultaneously in a Neubauer's haemocytometer.

© The Macmillan Press Ltd., 1985

The cell concentration was adjusted to the standard of $3 \times 10^6$ mononuclear cells $ml^{-1}$ for triplicates. Effusions were centrifuged at 2000 r.p.m. for 20 min. The resulting pellets were resuspended in McCoy's 5 A medium and pressed through a 25 $\mu$m mesh filter. Determination of total and viable cell count and adjustment to the standard cell concentration were executed as described above.

*Drug testing*

The following drugs, which were kindly provided by various companies, were used in our experiments: aclacinomycin A (Behring Werke, FRG); adriamycin (Farmitalia-Montedison, Italy); cisplatin (Bristol Myers Laboratories, USA); 5-fluorouracil (Hoffmann-La Roche Laboratories, Switzerland); methotrexate (EBEWE Laboratories, Austria, and Cyanamid Laboratories, USA); tamoxifen (ICI Laboratories, UK); vincristine (Eli Lilly Laboratories, USA).

Aliquots of the cell suspension were distributed over vials. The final concentration was adjusted to $10^6$ cells $ml^{-1}$ by adding medium only to the controls and to those vials taken for continuous exposure or by adding drug dilutions plus medium to the vials taken for 1 h drug exposure. Several drug concentrations achievable in man (Alberts *et al.*, 1980) of each chosen drug were tested on every tumour. After incubation of drug tests and controls at 37°C in a humidified 5.5% $CO_2$ atmosphere for 1 h – the time chosen for schedule independent and/or chemically and biologically unstable substances – samples were washed twice in medium and cultivated. Those drugs and hormones being schedule dependent and/or chemically and biologically stable were tested by exposing the cells to the drug dilutions for the entire incubation period (Ludwig & Alberts, 1984).

*Culture*

Aliquots of cells resuspended in 0.3% agar in enriched CMRL 1066 medium were plated in 35 mm petri dishes over an underlayer of 0.5% agar in enriched McCoy's 5 A medium. The final concentration was $5 \times 10^5$ cells per petri dish. After examination for clumps, the petri dishes were incubated in a humidified 5.5% $CO_2$ atmosphere at 37°C for 2 to 3 weeks. Control plates were routinely reexamined to assess the optimal time for study evaluation.

*Test evaluation*

Colonies were defined as aggregates of 40 or more cells originating from a single cell by cell division (Hamburger & Salmon, 1977). Evaluation of the drug studies was done counting established colonies under an inverted microscope. Petri dishes with 5 or more colonies were considered positive, but only cultures with 30 or more colonies in the controls were adequate for drug evaluation. Reduction of colony growth by 70% or more in the drug tests compared to the controls was considered to represent drug sensitivity.

*Identification of tumour cells*

Permanent slides of the established cultures prepared according to the method of Salmon & Buick (1979) were routinely compared with the cytocentrifuge slides made of each original cell suspension for morphologic evaluation and documentation. A nuclear grading system similar to Bloom's histologic grading (Bloom, 1950*a,b*) was used to obtain data enabling comparison between cytology and histology. Additional methods used for confirming the malignant nature of the cells from the original cell suspensions and from those growing as colonies were chromosome analysis (Trent, 1980) and in a few cases, transmission electron microscopy (Persky *et al.*, 1982).

*Statistical methods*

Comparisons between two groups of data which were not distributed normally were done using Wilcoxon tests. For correlation, the Spearman rank correlation was chosen. Frequencies were evaluated by $\chi^2$ tests.

### Results

Forty-nine out of 87 samples (56%) sent to our laboratory were accepted for evaluation. Reasons for exclusion of 38 specimens are detailed in Table I. Whereas all of the exclusions of body fluids were due to negative cytology, inadequate cell viability (<30% viable cells by the trypan blue exclusion method) was the main reason for biopsies being unsuitable for evaluation. The origin of the test material is described on Table II.

Clonal growth was observed in only 17/49 (35%) of all adequate breast cancer specimens; effusions providing sufficient growth in more than three quarters of cases, and biopsies in only one of four cases. The overall growth of effusions was significantly higher than that of biopsies ($P<0.01$, $\chi^2=10.04$). The median colony number of the effusions was 64 and that of the biopsies, 18. The mean cloning efficiency for all tests was 0.0111% (median 0.0092% range: 0.0010%–0.0486%; Table III).

With a median of 93%, the cell viability of the effusions (mean: 87%, range: 48–99%) was

**Table I** Criteria for exclusion of tumour specimens from evaluation

| | |
|---|---|
| Negative histologic/cytologic findings | 13/38 (35%) |
| Inadequate cell viability (<30%)[a] | 18/38 (46%) |
| Insufficient tumour material | 6/38 (16%) |
| Contamination | 1/38 (3%) |

[a]Cell viability was determined using the trypan blue dye exclusion method.

**Table II** Origin of test material

| *Biopsies* (*55%*) | *27/49* |
|---|---|
| Primary tumours | 22 |
| Local recurrences | 2 |
| Metastases | 3 |
| *Effusions* (*45%*) | *22/49* |
| Pleural effusions | 16 |
| Ascites | 4 |
| Pericardial effusions | 2 |

**Table III** Growth of breast cancer specimens in the HTCA

| | *Biopsies* | | | *Effusions* | | | *Total* | | |
|---|---|---|---|---|---|---|---|---|---|
| | *n* | *median* | *range* | *n* | *median* | *range* | *n* | *median* | *range* |
| Colony number | | | | | | | | | |
| G. overall[a] | 4 | 18 | 5–30 | 13 | 64 | 9–243 | 17 | 46 | 5–243 |
| G. sufficient[b] | 1 | — | — | 10 | 67 | 32–243 | 11 | 65 | 30–243 |
| Cloning efficiency[c] | | | | | | | | | |
| G.overall | 4 | 0.0035 | 0.0010–0.0060 | 13 | 0.0128 | 0.0018–0.0486 | 17 | 0.0092 | 0.0010–0.0486 |
| G. sufficient | 1 | — | — | 10 | 0.0133 | 0.0064–0.0486 | 11 | 0.0130 | 0.0060–0.0486 |

[a]G. (growth) overall comprises all grown specimens with ≧5 colonies per control plate.
[b]G. (growth) sufficient refers to grown specimens with ≧30 colonies per control plate.
[c]Cloning efficiency (%) $= \frac{\text{No. of colonies}}{\text{No. of mononuclear cells}} \times 100$.

significantly higher ($P<0.01$) than that of the biopsies which had a median of 55% (mean: 57%, range: 33–99%). The viability of the specimens providing colony growth was significantly higher than that of the non-growing samples ($P<0.01$). This dependence of growth on cell viability was underlined by the positive correlation between viability and cloning efficiency ($r_s=0.48$; $P<0.05$).

In the following section we attempt to examine colony growth in relation to established risk factors for women with breast cancer.

Although growth occurred more frequently from biopsies attained from advanced tumours, correlation with tumour stage was not significant. Furthermore, no association was found between clonal growth and the corresponding histopathologic grading in our specimens ($n=22$).

Regarding menopausal status no significant difference in growth or lack of growth was found between premenopausal ($n=11$) and postmenopausal ($n=32$) women in our study. In addition, hormone receptor levels of the tumours of 38 patients were determined. There seemed to be a negative relationship between progesterone receptor (PgR) level and growth, but this did not reach statistical significance ($P<0.1$) (Table IV).

Colony growth in the HTCA has been discussed as a possible risk factor for survival, at least regarding other malignancies (Mattox & Von Hoff, 1980; Durie *et al.,* 1983). In our material no association was found between *in vitro* growth of the tumour specimens and the survival of the corresponding patients.

**Table IV** Hormone receptor level-related growth of breast cancer specimens in the HTCA

| *Hormone receptor status* | | *Growth in the HTCA* | |
|---|---|---|---|
| $P_gR$[a] | $E_2R$[b] | *Positive*[c] | *Negative*[d] |
| + | + | 2 | 8 |
| + | – | 0 | 5 |
| – | + | 3 | 4 |
| – | – | 6 | 10 |

[a]Progesterone receptor level: +: ≧10 fmol mg$^{-1}$ cytosol protein, –: <10 fmol mg$^{-1}$ cytosol protein.
[b]Oestrogen receptor level: +: ≧10 fmol mg$^{-1}$ cytosol protein, –: <10 fmol mg$^{-1}$ cytosol protein
[c]≧5 colonies in the controls.
[d]5<5 colonies in the controls.

A total of 129 single drug tests could be performed on specimens from 14 different patients. *In vitro* test results of samples of patients treated by first or second line chemotherapy are illustrated separately in Figure 1. The single drug response rates *in vitro* corresponded well with the reported clinical single drug response rates in the case of

**Figure 1** Chemosensitivity of breast cancer specimens *in vitro* and reported equivalent monochemotherapy response rates *in vivo*. The percentage of sensitivity observed in the *in vitro* assay is illustrated by columns. Sensitivity of specimens of patients receiving first line therapy is shown in the dotted columns, that of samples from women under second line chemotherapy is demonstrated by the striped columns. The following drug concentrations ($C_{1,2}$) were used for testing: adriamycin (ADR): $C_1 = 0.1\,\mu g\,ml^{-1}$, $C_2 = 0.01\,\mu g\,ml^{-1}$; 5-fluorouracil (5-FU): $C_1 = 10.0\,\mu g\,ml^{-1}$, $C_2 = 1.0\,\mu g\,ml^{-1}$; methotrexate (MTX): $C_1 = 0.2\,\mu g\,ml^{-1}$, $C_2 = 0.01\,\mu g\,ml^{-1}$; vincristine (VCR): $C_1 = 0.05\,\mu g\,ml^{-1}$, $C_2 = 0.0005\,\mu g\,ml^{-1}$; aclacinomycin A (ACM): $C_1 = 0.1\,\mu g\,ml^{-1}$; cisplatin (DDP): $C_1 = 0.1\,\mu g\,ml^{-1}$, $C_2 = 0.01\,\mu g\,ml^{-1}$; tamoxifen (TAM): $C_1 = 10^{-6}\,M$. The numbers (*n*) of tests performed of the single items are indicated below the corresponding columns.

adriamycin and 5-fluorouracil, whereas in the case of methotrexate agreement for the first line therapy was found only at the lower *in vitro* drug level. Vincristine, aclacinomycin and cisplatin did not correspond well, neither at the higher nor at the lower test concentration. For most of the drugs the higher test concentration showed increased sensitivity rates *in vitro*. Monochemotherapy response rates for first line therapy (Hellman *et al.*, 1982; Kolaric & Roth, 1983; Oka, 1978) are represented by horizontal bars in Figure 1.

**Table V** Sensitivity of breast cancer specimens to tamoxifen with regard to their hormone receptor status

| $P_gR^a$ | $E_2R^b$ | $R^c$ | $S^d$ |
|---|---|---|---|
| + | + | 2 | 1 |
| + | – | 1 | 1 |
| – | + | 3 | 0 |
| – | – | 1 | 0 |

[a]$P_gR$ = progesterone receptor level: + = ≥10 fmol $mg^{-1}$ cytosol protein; – = <10 fmol $mg^{-1}$ cytosol protein.
[b]$E_2R$ = oestrogen receptor level: + = ≥10 fmol $mg^{-1}$ cytosol protein; – = <10 fmol $mg^{-1}$ cytosol protein.
[c]R = resistant: colony survival >30% of control count.
[d]S = sensitive: colony survival 30% of the control count or less.

The *in vitro* response rate of 15% for tamoxifen was very low with regard to the clinical results (McGuire *et al.*, 1977). In 9 of 13 established tumour specimens tested against tamoxifen we obtained the relevant hormone receptor levels, which showed an uncharacteristic distribution (Table V).

Hormone receptor level-related chemosensitivity is demonstrated on Table VI. Resistance to various

**Table VI** Chemosensitivity in relation to hormone receptor status in breast cancer

| $E_2R^d$ | $R_gR^e$ | $N^a$ | $R^b$(%) | $S^c$(%) |
|---|---|---|---|---|
| + | + | 24 | 22 (92) | 2 (8) |
| + | – | 29 | 29 (100) | 0 (0) |
| – | + | 14 | 9 (64) | 5 (36) |
| – | – | 16 | 2 (12) | 14 (88) |

[a]No. of single drug tests.
[b]R = resistant: colony survival >30% of the control count.
[c]S = sensitive: colony survival 30% of the control count or less.
[d]$E_2R$ = oestrogen receptor level: + = ≥10 fmol $mg^{-1}$ cytosol protein; – = <10 fmol $mg^{-1}$ cytosol protein.
[e]$P_gR$ = progesterone receptor level: + = ≥10 fmol $mg^{-1}$ cytosol protein: – = <10 fmol $mg^{-1}$ cytosol protein.

cytotoxic drugs was found in 92% and 100% of the oestrogen receptor ($E_2R$) positive, but only in 12% and 64% of the $E_2R$ negative samples ($P<0.01$).

Comparison between *in vitro* prediction and clinical course could be made in 6 patients only. In all other cases, the cytotoxic drugs tested were not the same as those given as therapy. In 4/4 cases resistance prediction was correct, and in 2/2 cases sensitivity was accurately predicted.

### Discussion

There are enormous discrepancies in most of the scant data on the use of HTCA in breast cancer sensitivity testing. Many of the differences are the result of varying definitions and interpretations of the fundamental notions about the assay system itself. Our results will be discussed relative to those reported in literature.

Even if our median colony number of 46 is quite comparable to that of Sandbach *et al.* (1982), who reported on 225 patients, and even if our percentage of sufficient growth (22%) appears equivalent to that of Jones *et al.* (1984) (27%), we have to take into consideration that similar values may mirror divergent results. The data reported by us are based on the definition of a colony as an aggregate of 40 or more cells originating from a single cell by replication (Hamburger & Salmon, 1977). Other reports on this topic are based on four different definitions of a colony – ranging from a minimum of 20 to a minimum of 50 cells originating from a single tumour cell (Kern *et al.*, 1982; Sutherland *et al.*, 1983; Touzet *et al.*, 1982; Sandbach *et al.*, 1982). The discrepancies in growth obtained by different authors are less surprising when initial 'standardized' materials are compared. Cloning efficiencies may relate to nucleated cells only (Jones *et al.*, 1984; Sutherland *et al.*, 1983; own results), to viable cells (Bernard *et al.*, 1983; Sandbach *et al.*, 1982), or even to viable tumour cells (Schlag *et al.*, 1982; Kern *et al.*, 1982).

The continuous search for parameters which could assist in the accurate prediction of prognosis led to the establishment of the TNM system. Sutherland *et al.* (1983) found no correlation between the TNM classification and the number of colonies grown in the HTCA. The higher frequency of growth among the tumours of more advanced TNM-stages in our study did not reach significance either. Histologic and cytologic grading are other means of surveying the aggressiveness of a neoplasm. In accordance with Benard *et al.* (1983) we found no association between the histopathologic grading and clonal growth in our material. The data contrast markedly with the observation made by Touzet *et al.* (1982), who did find a significant positive correlation between colony growth *in vitro* and tumour grade. Furthermore colony growth *in vitro* seems to be independent of the menopausal status.

Another acknowledged prognostic factor for breast cancer patients is hormone receptor status. In contrast to several authors who found no correlation between the number of colonies and the $E_2R$ level (Sutherland *et al.*, 1983) or between *in vitro* colony growth and the $E_2R$ level (Sandbach *et al.*, 1982; Benard *et al.*, 1983), we found a negative trend between $P_gR$ positive tumours and their growth in culture ($P<0.1$). The absence of the differentiation product, $P_gR$ may be considered an indicator of shorter disease-free survival (Clark *et al.*, 1983) caused by more primitive cancer cells. Their aggressiveness seems to be reflected by their growth in the double layer agar system.

Additionally, colony growth *in vitro* has been shown to represent an independent factor indicative of patients' survival (Mattox & Von Hoff, 1980; Durie *et al.*, 1983). We found no correlation between colony growth *in vitro* and patients' survival in our study. Both Sutherland *et al.* (1983) and Benard *et al.* (1983) found such relations, at least for subgroups of patients.

The original aim of the creation of the HTCA was to develop a tool enabling therapists to predict *in vitro* sensitivity or resistance of an individual patient's tumours to a cytotoxic drug. We found that 36% of untreated tumours and 13% of pretreated tumours responded to single agent chemotherapy *in vitro*, assuming a colony kill of 70% or more as sensitive. These numbers correspond well to the overall percentages in patients under the same monochemotherapy *in vivo* (Figure 1).

In our study, we did not observe the increase in sensitivity to tamoxifen which had been expected from estimates of hormone receptor concentration (Jakesz *et al.*, In press). In contrast, we found a numerical increase in tamoxifen-resistant specimens among the $E_2R$ positive ones. There are no data indicating that the HTCA is equivalent to the determination of hormone receptor levels for the prediction of tumour sensitivity to hormones. To date, there are insufficient data from which to derive a consensus on the value of hormone receptor determination for the prediction of chemosensitivity in women with breast cancer (Lippmann *et al.*, 1978; Kiang *et al.*, 1978; Dittrich *et al.*, 1980). The *in vitro* data of this study demonstrate that positive $E_2R$ levels are correlated with resistance to cytotoxic drugs to a statistically significant degree ($P<0.01$), thus confirming Lippman's hypothesis of increasing chemosensitivity of $E_2R$ negative tumours. These data should be the subject of further studies.

The only parameter for ascertaining the value of an *in vitro* predictive system is the congruence between accuracy of *in vitro* prediction and clinical course of patients treated accordingly. Information on this question is scant to date. Sutherland *et al.* (1983) reported on two correct resistance predictions; Jones *et al.* (1984) described a total of 59 correlations, 22 thereof having been correct resistance predictions for single agent chemotherapy, and 13 correct resistance predictions for combination chemotherapy. We were able to do 6 *in vitro–in vivo* correlations; in 4 cases resistance and in 2 cases, sensitivity were correctly predicted.

In our study, we tried to establish correlations between factors inherent in patients with breast cancer, such as tumour size, histopathologic grading, hormone receptor level, menopausal status, chemo- and hormone-sensitivity, as well as survival prognosis on the one hand, and the behaviour of these patients' tumour cells in the soft agar culture on the other. Because of the low overall number of results obtained by this test system at present, any deployment of the HTCA with the intention of evaluating chemo- and hormone-sensitivity or of predicting the prognosis of women with breast cancer must still be considered investigative. Attention must be focussed on improving the test system before the HTCA may be expected to provide further essential information on the management of patients with breast cancer.

Supported by a grant from the 'Kommission Onkologie der Medizinischen Fakultät der Universität Wien' and the 'Anton Dreher-Gedächtnisschenkung für medizinische Forschung'.

We thank Mrs Margit Koller for her competent secretarial work and Mrs Diana Reese-Soltész for her suggestions.

### References

ALBERTS, D.S., CHEN, H.S.G. & SALMON, S.E. (1980). *In vitro* drug assay: Pharmacologic considerations. In *Cloning of Human Tumor Stem Cells*, p. 197 (ed. Salmon). Liss: New York.

BENARD, J., DA SILVA, J. & RIOU, G. (1983). Culture of clonogenic cells from various human tumours drug sensitivity assay. *Eur. J. Cancer Clin. Oncol.*, **19**, 65.

BLOOM, H.J.G. (1950*a*). Prognosis in carcinoma of the breast. *Br. J. Cancer*, **4**, 259.

BLOOM, H.J.G. (1950*b*). Further studies on prognosis of breast carcinoma. *Br. J. Cancer*, **4**, 37.

CLARK, G.M., MCGUIRE, W.L., HUBAY, Ch.A., PEARSON, O.H. & MARSHALL, J.S. (1983). Progesterone receptors as a prognostic factor in stage II breast cancer. *N. Engl. J. Med.*, **309**, 1343.

DITTRICH, Ch., BETTELHEIM, P., BIEGELMAYER, C. & 10 others. (1980). Prognostic significance of estrogen receptors. In *Diagnosis and Treatment of Breast Cancer*, p. 111 (eds. Lewison & Montague). Williams & Wilkins: Baltimore–London.

DITTRICH, Ch. (1984). *In vitro*-Chemosensitivitätstestung mit dem Human Tumor Stem Cell Assay (HTSCA) beim Mammakarzinom. *Wien. Klin. Wochenschr.*, **13**, 508.

DITTRICH, Ch., SATTELHAK, E., JAKESZ, R. & 8 others. (1984). Testing of mammary cancer in the human tumor stem cell assay. In *Human Tumor Cloning*, p. 551 (eds. Salmon & Trent). Grune & Stratton: Orlando.

DURIE, B.G.M., YOUNG, L.A. & SALMON, S.E. (1983). Human myeloma *in vitro* colony growth: Interrelationship between drug sensitivity, cell kinetics, and patient survival duration. *Blood*, **61**, 929.

HAMBURGER, A.W. & SALMON, S.E. (1977). Primary bioassay of human tumor stem cells. *Science*, **197**, 461.

HELLMAN, S., HARRIS, J.R., CANELLOS, G.P. & FISCHER, G. (1982). Cancer of the breast. In *Cancer, Principles and Practice of Oncology*, p. 950 (eds. De Vita *et al.*). Lippincott: Philadelphia–Toronto.

HILF, R., FELDSTEIN, M.L., GIBSON, S.L. & SAVLOV, E.D. (1980). The importance of estrogen receptor analysis as a prognostic factor for recurrence and response to chemotherapy in women with breast cancer. *Cancer*, **45**, 1983.

JAKESZ, R., DITTRICH, Ch., HAVELEC, L. & 7 others. (1984). Die Bedeutung der Kolonienbildung im Human Tumor Cloning Assay zur Erfassung der Chemosensitivität beim Mammakarzinom (in press).

JONES, St.E., DEAN, J.C. & SALMON, S.E. (1984). The clinical usefulness of the human tumor clonogenic assay (HTSCA) in breast cancer. In *Human Tumor Cloning*, p. 557 (eds. Salmon & Trent). Grune & Stratton: Orlando.

KERN, D.H., CAMPBELL, M.A., COCHRAN, A.J., BURK, M.W. & MORTON, D.L. (1982). Cloning of human solid tumors in soft agar. *Int. J. Cancer*, **30**, 725.

KIANG, D.T., PRENNING, D.H., GOLDMANN, A.I., ASCANSAO, V.E. & KENNEDY, B.J. (1978). ER and response to chemotherapy and hormonal therapy in advanced breast cancer. *N. Engl. J. Med.*, **299**, 24.

KOLARIC, K. & ROTH, A. (1983). Phase II clinical trial of cis-dichlorodiammine-platinum (cis-DDP) for antitumorigenic activity in previously untreated patients with metastatic breast cancer. *Cancer Chemother. Pharmacol.*, **11**, 108.

LIPPMAN, M.E., ALLEGRA, J.C., THOMPSON, E.B. & 7 others. (1978). The relation between estrogen receptors and response rate to cytotoxic chemotherapy in metastatic breast cancer. *N. Engl. J. Med.*, *298*, 1223.

LUDWIG, R. & ALBERTS, D.S. (1984). Chemical and biological stability of anticancer drugs used in a human tumor clonogenic assay. *Cancer Chemother. Pharmacol.*, **12**, 142.

MATTOX, D.E. & VON HOFF, D.D. (1980). *In vitro* stem cell assay in head and neck squamous carcinoma. *Amer. J. Surg.*, **140**, 527.

McGUIRE, W., HORWITZ, K.B., PEARSON, O.H. & SEGALOFF, A. (1977). Current status of estrogen and progesterone receptors in breast cancer. *Cancer*, **39**, 2934.

OKA, S. (1978). A review of clinical studies on aclacinomycin A – phase I and preliminary phase II evaluation of ACM. *Sci. Rep. Res. Inst. Tohoku Univ.* C, **25**, 37.

OSBORNE, C., YOCHMOWITZ, M., KNIGHT, W. & McGUIRE, W. (1980). The value of estrogen and progesterone receptors in the treatment of breast cancer. *Cancer*, **40**, 2884.

PARIDAENS, R., SYLVESTER, R., FERRAZZI, E., LEGROS, N., LECLERQ, G. & HENSON, J. (1980). Clinical significance of the quantitative assessment of estrogen receptors in advanced breast cancer. *Cancer*, **36**, 2889.

PAVELIC, Z.P., SLOCUM, H.K., RUSTUM, Y.M. & 5 others. (1980). Growth of cell colonies in soft agar from biopsies of different human solid tumors. *Cancer Res.*, **40**, 4151.

PERSKY, B., THOMSON, S.P., MEYSKENS, F.L. JR. & HENDRIX, M.J.C. (1982). Methods for evaluating the morphological and immunohistochemical properties of human tumor colonies grown in soft agar. *In vitro*, **18**, 929.

SALMON, S.E., HAMBURGER, A.W., SOEHNLEN, B., DURIE, B.G.M., ALBERTS, D.S. & MOON, T.E. (1978). Quantitation of differential sensitivity of human tumor stem cells to anticancer drugs. *N. Engl. J. Med.*, **298**, 1321.

SALMON, S.E. & BUICK, R.N. (1979). Preparation of permanent slides of intact soft-agar colony cultures of hematopoietic and tumor stem cells. *Cancer Res.*, **39**, 1133.

SANDBACH, J., VON HOFF, D.D., CLARK, G., CRUZ, A.B., O'BRIEN, M. & THE SOUTH CENTRAL TEXAS HUMAN TUMOR CLONING GROUP. (1982). Direct cloning of human breast cancer in soft agar culture. *Cancer*, **50**, 1315.

SCHLAG, P., WOLFRUM, J., VERGANI, G., SCHREHL, W. & HERFARTH, Ch. (1982). Wachstum von Tumorzellkolonien bei menschlichen soliden *Tumoren. Dtsch. Med. Wochenschr.*, **107**, 1173.

SUTHERLAND, C.M., MATHER, F.J., CARTER, R.D., CERISE, E.J. & KREMENTZ, E.T. (1983). Breast cancer as analyzed by the human tumor stem cell assay. *Surgery*, **94**, 370.

TOUZET, Cl., RUSE, F., CHASSAGNE, J. & 5 others. (1982). *In vitro* cloning of human breast tumor stem cells: Influence of histological grade on the success of cultures. *Br. J. Cancer*, **46**, 668.

TOUZET, Cl., RUSE, F., FERRIERE, J.P. & 5 others (1983). Clonage *in vitro* de cellules souches tumorales humaines. *Pathol. Biol.*, **31**, 33.

TRENT, J.M. (1980). Cytogenetic analysis of human tumor cells cloned in agar. In: *Cloning of Human Tumor Stem Cells*, p. 165. (Ed. Salmon). Liss: New York.